# Transcriptome-Based Identification of the SaR2R3-MYB Gene Family in *Sophora alopecuroides* and Function Analysis of SaR2R3-MYB15 in Salt Stress Tolerance

**DOI:** 10.3390/plants13050586

**Published:** 2024-02-21

**Authors:** Yuan Wang, Xiaoming Yang, Yongning Hu, Xinqian Liu, Tuya Shareng, Gongxiang Cao, Yukun Xing, Yuewen Yang, Yinxiang Li, Weili Huang, Zhibo Wang, Gaowa Bai, Yuanyuan Ji, Yuzhi Wang

**Affiliations:** 1 Inner Mongolia Academy of Forestry Science, Hohhot 010021, China; 2 Co-Innovation Center for Sustainable Forestry in Southern China, Nanjing Forestry University, Nanjing 210037, China; 3 Inner Mongolia Engineering Laboratory of Economic Forest Sterile Virus-Free Cultivation, Hohhot 010021, China; 4 Inner Mongolia Ordos Forest Ecosystem Research Station, Ordos 016100, China

**Keywords:** *Sophora alopecuroides*, R2R3-MYB, expression pattern, gene function, salt stress

## Abstract

As one of the most prominent gene families, R2R3-MYB transcription factors significantly regulate biochemical and physiological processes under salt stress. However, in *Sophora alopecuroides*, a perennial herb known for its exceptional saline alkali resistance, the comprehensive identification and characterization of SaR2R3-MYB genes and their potential functions in response to salt stress have yet to be determined. We investigated the expression profiles and biological functions of SaR2R3-MYB transcription factors in response to salt stress, utilizing a transcriptome-wide mining method. Our analysis identified 28 SaR2R3-MYB transcription factors, all sharing a highly conserved R2R3 domain, which were further divided into 28 subgroups through phylogenetic analysis. Some SaR2R3-MYB transcription factors showed induction under salt stress, with SaR2R3-MYB15 emerging as a potential regulator based on analysis of the protein–protein interaction network. Validation revealed the transcriptional activity and nuclear localization of SaR2R3-MYB15. Remarkably, overexpression of *SaR2R3-MYB15* in transgenic plants could increase the activity of antioxidant enzymes and the accumulation of proline but decrease the content of malondialdehyde (MDA), compared with wild-type plants. Moreover, several salt stress-related genes showed higher expression levels in transgenic plants, implying their potential to enhance salt tolerance. Our findings shed light on the role of SaR2R3-MYB genes in salt tolerance in *S. alopecuroides*.

## 1. Introduction

Transcription factors (TFs) cooperate with *cis*-elements to regulate the expression of specific genes in response to various biological processes, shaping plants themselves and enhancing their adaptation to diverse environments [1]. The MYB transcription factors are distinguished by the presence of one to four repeats of ~50 amino acids, which form three helices [2]. The MYB transcription factors are classified into four typical family members: R2R3-MYB, MYB-related, R1R2R3-MYB, and 4R-MYB proteins [3]. The hydrophobic core of the “helix-turn-helix” shape, comprising three tryptophan residues on the second and third helices, is believed to play a role in recognition when binding to a condensed DNA sequence [4]. Since the first discovery of the involvement of R2R3-MYB transcription factors in maize anthocyanin biosynthesis, an increasing number of plant R2R3-MYB genes have been systematically explored, making them one of the most extensively studied gene families in plants [5]. The R2R3-MYB transcription factors generally exhibit various functions due to their relatively conserved DNA-binding and transcriptional regulatory domains. The identification of R2R3-MYB transcription factors has significantly increased across a diverse range of species, including 155 in *Oryza sativa* [6], 126 in *Arabidopsis thaliana* [4], and 245 in *Helianthus annuus* [7]. The MYB transcription factors have rapidly expanded [3] and play crucial roles in plant development, stress responses, and various biochemical and physiological activities [2,5].

Salinity, as an adverse environmental factor, affects more than 6% of the world’s land area [8]. Salt stress leads to ionic stress, osmotic stress, and secondary stresses, particularly oxidative stress, which impede plant development and productivity [9]. In response to salt stress, plants must modify their physiological and biochemical mechanisms to mitigate stress damage, maintain ionic and osmotic balance, and promote repair processes [8,10]. Several MYB transcription factors have been found to play a role in plants’ salt stress response, and their downstream targets have been identified [11]. For example, in *Arabidopsis*, overexpression of *AtMYB49* enhances salt stress tolerance by interacting with the promoters of *MYB41*, *ASFT*, *FACT*, and *CYP86B1* [12]. Moreover, *AtMYB20* negatively inhibits the expression of protein phosphatase 2Cs (PP2Cs), which is the key negative regulator of abscisic acid (ABA) signaling. This inhibition leads to improved salt tolerance in plants [13]. Additionally, salt tolerance is positively influenced by *AtMYB42*, which controls the expression of SOS2 (salt excessively sensitive 2) in *Arabidopsis* [14]. In rice, the expression of *OsMYB91* is induced under salt stress conditions, resulting in increased expression of *SLR1* and enhanced accumulation of ABA. This, in turn, promotes plant development and improves tolerance to salt stress [15].

*Sophora alopecuroides* L. (Leguminosae), commonly known as “Kudouzi” in China, is a perennial medicinal herb with remarkable drought and saline alkali resistance. It is primarily distributed in Western and Central Asia, particularly in the Loess Plateau, China [16,17]. In Northwest China, *S. alopecuroides* plays a crucial role in environmental conservation owing to its well-developed root system, which confers excellent abiotic stress tolerance and anti-sandstorm performance [18]. Numerous biologically active compounds, including alkaloids, steroids, polysaccharides, and flavonoids, have been successfully identified in *S. alopecuroides* [19]. The plant has garnered attention in the medical field for its anti-inflammatory, anti-tumo, and antibacterial effects [5,19]. The roots and seeds of *S. alopecuroides* are extensively utilized as antibiotics, painkillers, and fever reducers [18]. Additionally, *S. alopecuroides* has exhibited significant resistance to salt stress, making it a valuable subject for stress resistance research [20]. Although the whole genome of *S. alopecuroides* is not yet available, several valuable transcriptome sequencing studies have investigated its response to salt stress [16,20,21]. However, the molecular mechanisms underlying the plant’s response to salt stress remain poorly understood, and little is known about the involvement of SaR2R3-MYB TFs in salt stress regulation in *S. alopecuroides*. 

This study utilized high-quality transcriptome databases to identify 28 putative full-length R2R3-MYB transcription factors in *S. alopecuroides* responsive to salt stress. Our investigation focused on classifying these transcription factors, examining their gene structure, and exploring their evolutionary relationships. To gain insights into their roles, we employed phylogenetic analysis and profiled expression patterns. Additionally, we explored the potential function of the salt-induced *SaR2R3-MYB15* through the analysis of subcellular localization, transcriptional activity, and genetic transformation. Our findings have established a solid foundation for future functional studies on R2R3-MYB transcription factors in *S. alopecuroides* and they provide valuable guidance for the molecular biotechnology-based selection of candidate genes in *S. alopecuroides* breeding programs.

## 2. Results

### 2.1. Identification and Physicochemical Properties of SaR2R3-MYB TFs

**Figure 1:**
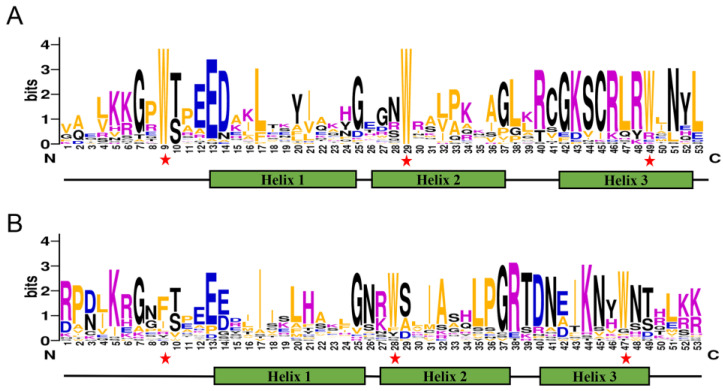
Analysis of conserved motif elements of SaR2R3-MYB transcription factors in *S. alopecuroides*. Sequence logos of R2 (**A**) and R3 (**B**) MYB repeats based on full-length alignments of all SaR2R3-MYB proteins. The position of the three α-helix (Helix 1–3) was marked. Bit score and red asterisks indicate the information content of each position in the sequence and the highly conserved tryptophan residues (Trp, W) in the MYB domain, respectively.

After assembling the transcriptome data, a total of 28 SaR2R3-MYB TFs were identified based on the presence of the complete R2R3-MYB domain (Table S1). To determine the conserved regions, we conducted multiple sequence alignment analyses. The results revealed that the R2 repeat contained three conserved tryptophan (Trp) residues, while the R3 domain only possessed the second and third Trp residues, as the first Trp was substituted by either isoleucine (Ile) or phenylalanine (Phe) (Figure 1). In addition to the DNA-binding domain, notable differences in the amino acid composition and length were observed.

The properties of SaR2R3-MYB proteins, including their protein isoelectric point (PI), subcellular localization, and molecular weight (MW), were analyzed (Table S1). The MW values of these proteins ranged from 12,346.42 Da (SaR2R3-MYB15) to 84,473.54 Da (SaR2R3-MYB18). The PI values varied from 4.48 (SaR2R3-MYB18) to 9.18 (SaR2R3-MYB15). All R2R3-MYB proteins were hydrophilic, with protein average hydrophilicity (GRAVY) prediction scores ranging from −0.993 (SaR2R3-MYB6) to −0.491 (SaR2R3-MYB5). In terms of protein length, SaR2R3-MYB18 was the longest predicted protein with 760 amino acids, while SaR2R3-MYB15 was the smallest with 105 amino acids. In terms of subcellular localization, SaR2R3-MYB16 was predicted to be located in the extracellular space, while SaR2R3-MYB23 was predicted to be located in the endomembrane. The other 26 SaR2R3-MYB proteins (83.33%) were all predicted to be located in the nucleus.

### 2.2. Conserved and Phylogenetic Classification of SaR2R3-MYB TFs

**Figure 2:**
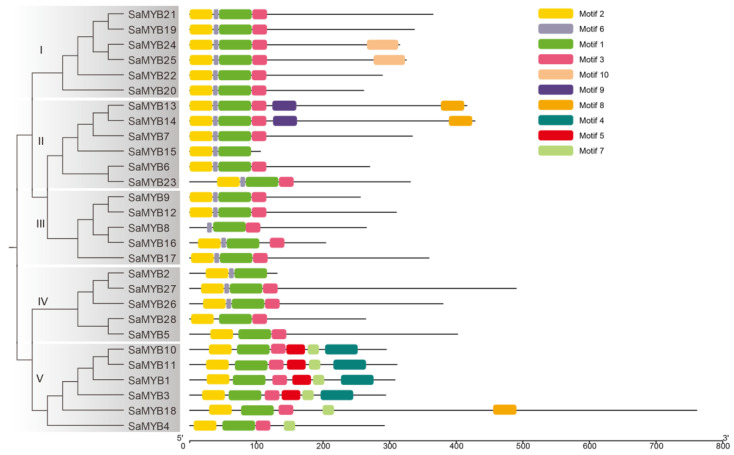
The phylogenetic tree was constructed with full-length sequences of SaR2R3-MYB proteins in *S. alopecuroides*. The architecture of the conserved motifs is characterized by different colored blocks.

The six subgroups of SaR2R3-MYB proteins with the full R2R3 structural domain showed that the SaR2R3-MYB TFs in *S. alopecuroides* belonged to different and separate clades (Figure 2). A total of 10 conserved motifs, labelled from 1 to 10, were identified in SaR2R3-MYB proteins (Figure 2). Notably, motifs 1 and 2 were present in every SaR2R3-MYB protein, and some of the SaR2R3-MYB proteins also shared motifs 3 and 6. Our research also showed that SaR2R3-MYB proteins in the same subgroup tended to possess similar motif compositions, suggesting their functional similarity.

**Figure 3:**
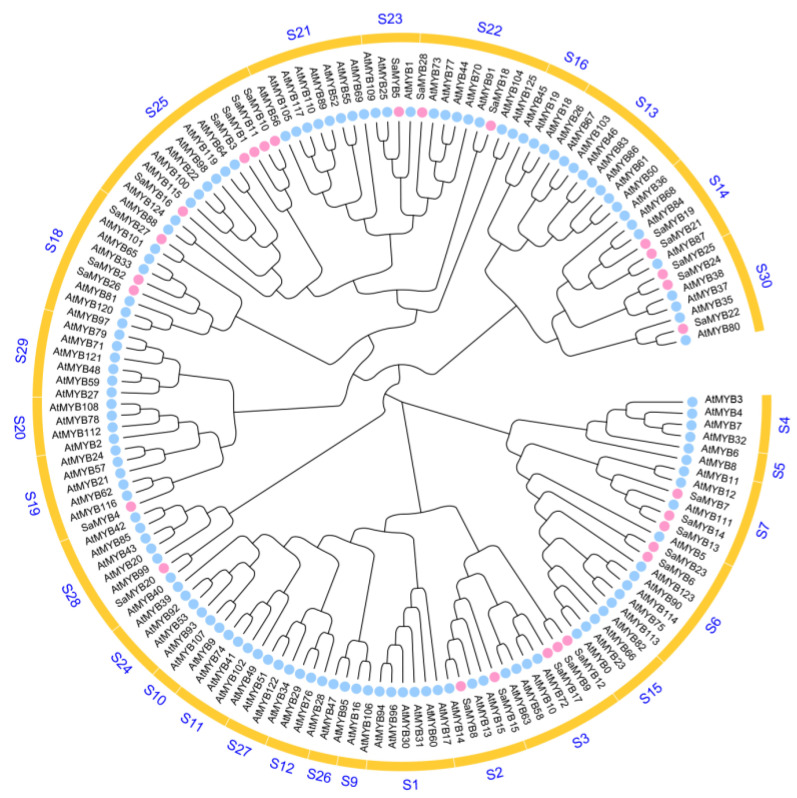
The phylogenetic tree of *R2R3-MYB* genes from *S. alopecuroides* and *Arabidopsis*. The pink and blue circles represent *SaR2R3-MYB* and *AtR2R3-MYB* genes, respectively. Each subgroup ID number was in the outer circle of the phylogenetic tree.

To unveil the evolutionary relationships among SaR2R3-MYB members, an unrooted phylogenetic tree was constructed with 169 AtR2R3-MYB members from *Arabidopsis* and 28 SaR2R3-MYB members from *S. alopecuroides* (Figure 3). All *R2R3-MYB* genes from both species were divided into 28 subgroups, labelled S1 to S25, in accordance with previous principles [3]. Several subgroups showed that SaR2R3-MYB members clustered with their *Arabidopsis* counterparts. The rest of the R2R3-MYB TFs from both species were named S26 to S30. The largest subgroup, S25, had 13 SaR2R3-MYB members. Five of them came from *S. alopecuroides*, and the other eight came from *Arabidopsis*. Interestingly, 16 subgroups of genes (S1, S4, S5, S9, S12, S10, S11, S13, S15, S16, S20, S21, S24, S26, S27, and S29) were specific to *Arabidopsis* and not present in *S. alopecuroides*.

### 2.3. Protein–Protein Interaction Network Predictions among SaR2R3-MYB Proteins

**Figure 4:**
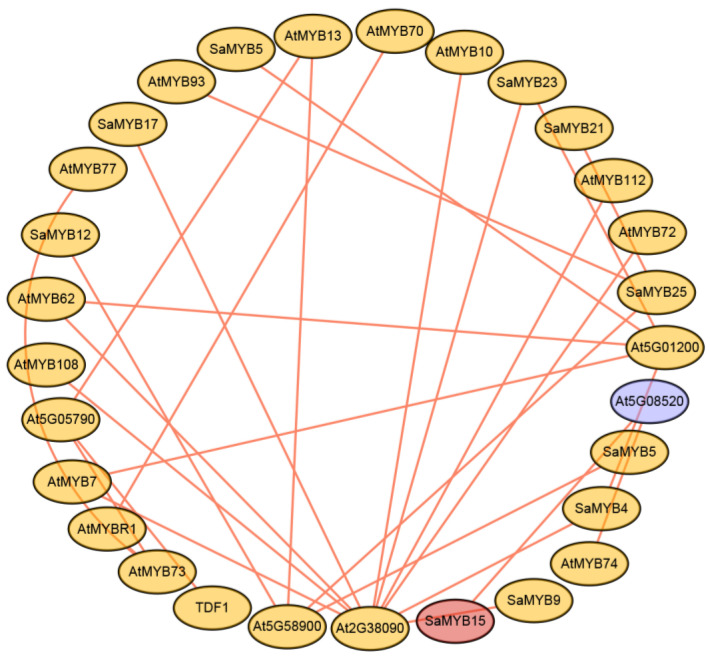
Protein–protein interaction analysis of SaR2R3-MYB gene family members in *S. alopecuroides* based on their orthologs in *Arabidopsis*. The red point represents the SaR2R3-MYB15 protein, and the purple cycle represents the putative protein (At5G08520) that interacted with the SaR2R3-MYB15 protein.

The protein–protein interaction network showed that most of the SaR2R3-MYB proteins interacted with one other, and several important interactions were predicted (Figure 4 and Table S3). Based on gene interaction relationships and function annotations, we identified critical proteins within this network that significantly influenced metabolic processes, tissue development, and biosynthetic processes in plants. Our study identified 10 SaR2R3-MYB proteins and 30 interacting protein branches in the network. Among these, two genes, AT2G38090 and AT5G01200 which belonged to the duplicated homeodomain-like superfamily proteins, exhibited close relationships with both SaR2R3-MYB and AtR2R3-MYB TFs. Previous research has suggested that AtMYB15 is a putative transcription factor involved in salt tolerance by upregulating the expression of stress-protective protein-coding genes [22]. Our study found that SaR2R3-MYB15 shared a high sequence homology with AtMYB15, implying a similar function. Moreover, SaR2R3-MYB15 showed a close relationship with the duplicated homeodomain-like superfamily protein (At5G08520). Similarly, AtR2R3-MYB74, which is involved in salt stress response, exhibited a strong association with the gene (At5g08520). Consequently, these transcription factors (AtR2R3-MYB15 and AtR2R3-MYB74) could participate in salt stress responses by interacting with the putative gene (At5g08520) in response to salt stress. While our anticipated network provided valuable insights for functional research, it is essential to note that further experimental confirmation is necessary.

### 2.4. Expression Patterns of SaR2R3-MYB under Salt Stress

**Figure 5:**
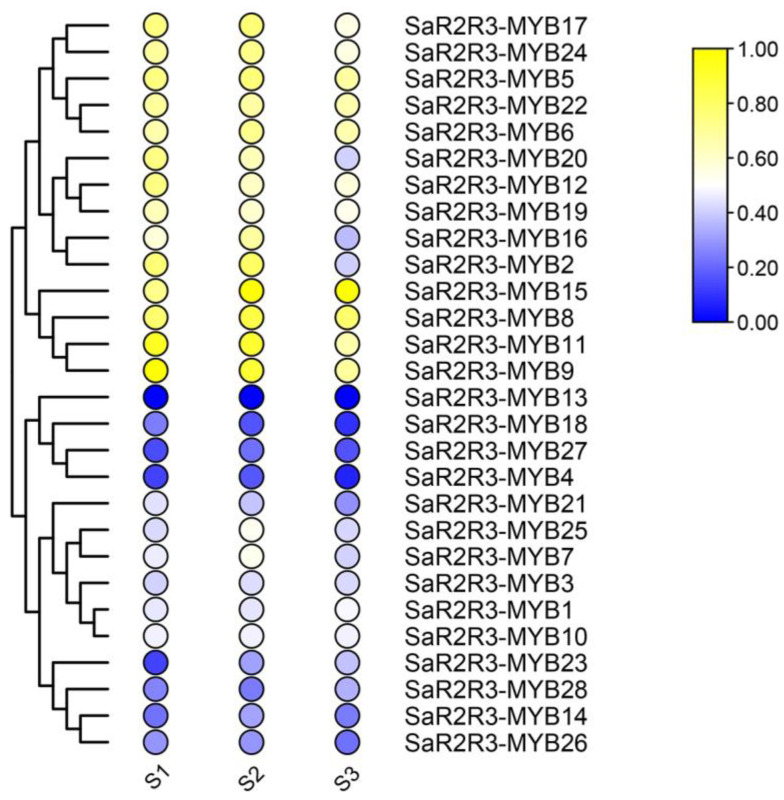
The expression patterns of *SaR2R3-MYB* genes were based on the transcriptome at different time points under salt stress. S1, S2, and S3 represent 0, 3, and 7 days, respectively.

To determine the expression patterns of the *SaR2R3-MYB* genes, we measured their transcript abundance at various time points under salt stress conditions. The hierarchical cluster was constructed based on the expression of *SaR2R3-MYB* genes to illustrate the various expression patterns of numerous genes (Figure 5). The expression of 16 *SaR2R3-MYB* genes was comparatively low at 0 days (S1) but high at 7 days (S3) under the 150 mM NaCl-induced salt stress, showing that these genes were upregulated in response to salt stress and served as potential candidates involved in the salt stress response. Conversely, ten members of the SaR2R3-MYB family displayed high expression levels at 0 days (S1) but low expression levels at 7 days (S3). Among these high expression levels of *SaR2R3-MYB* genes, the expression trend of *SaR2R3-MYB15* and *SaR2R3-MYB23* showed a steady increase as the duration of salt stress increased. Interestingly, the expression of these two genes (*SaR2R3-MYB15* and *SaR2R3-MYB23*) increased tenfold after 7 days (S3) of salt stress compared to 0 days (S1).

Furthermore, we used qRT–PCR to assess the expression levels of all *SaR2R3-MYB* genes. Our findings indicated that most genes had identical expression patterns to those evaluated by transcriptome (Figure S1). Notably, the expression levels of the *SaR2R3-MYB15* and *SaR2R3-MYB23* genes under salt stress were considerably higher at 7 days (S3) than at 0 days (S1) and 3 days (S2), suggesting that these two genes likely participate in the response to salt stress.

### 2.5. Subcellular Localization and Transcriptional Activity of SaR2R3-MYB15

**Figure 6:**
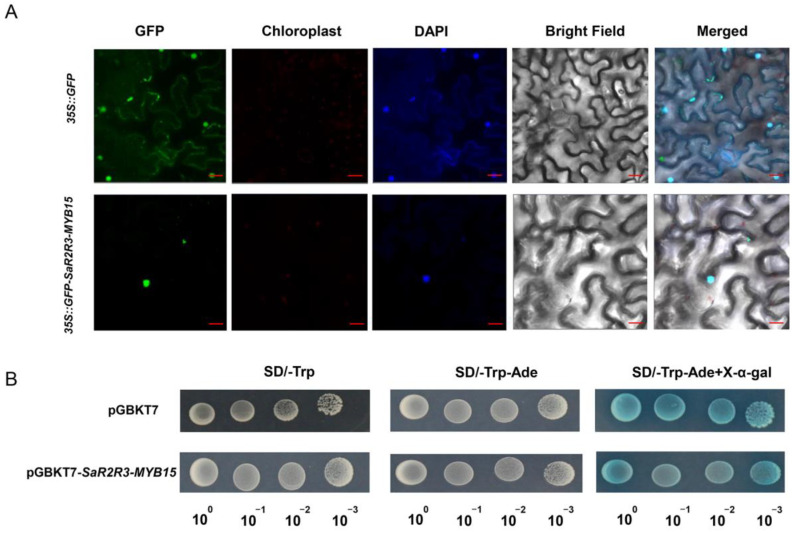
Subcellular localization and transactivation analysis of the SaR2R3-MYB15 protein. The GFP-fused protein of SaR2R3-MYB15 and free GFP proteins were transiently expressed in tobacco leaves; the scale bars represent 20 μm (**A**). The SaR2R3-MYB15 fusion construct and the positive construct were incubated in the SD/-Trp, SD/-Trp-Ade, and SD/-Trp-Ade+X-α-gal media (**B**).

To shed light on its potential role in transcriptional control mechanisms, the subcellular localization of SaR2R3-MYB15 (35S::SaR2R3-MYB15-GFP) was explored by transferring the *SaR2R3-MYB15* gene to tobacco leaves. Our findings revealed that the SaR2R3-MYB15 protein was located in the nucleus, where GFP fluorescence of 35S::SaR2R3-MYB15-GFP appeared (Figure 6A).

The development and growth status of single yeast colonies on a nutrient-deficient medium were used to adjust the transcription activity of SaR2R3-MYB15. The pGBKT7-SaR2R3-MYB15 recombinant plasmid transformed into a yeast strain that grew well on SD/-Trp and SD/-Trp-Ade (Figure 6B). Blue colonies of the positive control and pGNKT7-SaR2R3-MYB15 yeast cells were observed on SD/-Trp-Ade + X-gal selective media, indicating that SaR2R3-MYB15 has a potential transcriptional activation function.

### 2.6. Malondialdehyde (MDA) Concentration Evaluation and the Transient Expression of SaR2R3-MYB15 in N. benthamiana

The transient transformation of *SaR2R3-MYB15* in tobacco resulted in a decrease in MDA concentration under salt stress compared to the control plants, indicating that the cell membrane of tobacco infected with pCAMBI1301-SaR2R3-MYB15 incurred less damage and exhibited improved salt tolerance (Figure S2A).

**Figure 7:**
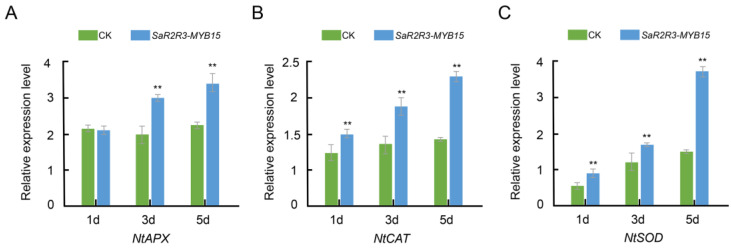
The relative expression level of ROS-related genes including *NbAPX* (**A**), *NbCAT* (**B**), and *NbSOD* (**C**) in 4-week-old tobacco plants under salt treatment after 1, 3, and 5 days. Student’s test was used to assess significant differences, and the standard errors are shown as the value of the error bars (**, *p* < 0.01).

Additionally, the qRT–PCR analysis revealed higher expression levels of *SaR2R3-MYB15* in transgenic tobacco plants (Figure S2B). To further explore the function of *SaR2R3-MYB15*, the expression patterns of three ROS-related genes, namely, *NbSOD, NbCAT*, and *NbAPX*, were investigated. Notably, tobacco plants overexpressing *SaR2R3-MYB15* displayed significantly elevated expression levels of *NbAPX*, *NbCAT*, and *NbSOD* compared to the control plants (Figure 7).

### 2.7. Overexpression of SaR2R3-MYB15 Improved the Tolerance of Transgenic Arabidopsis to Salt Stress

**Figure 8:**
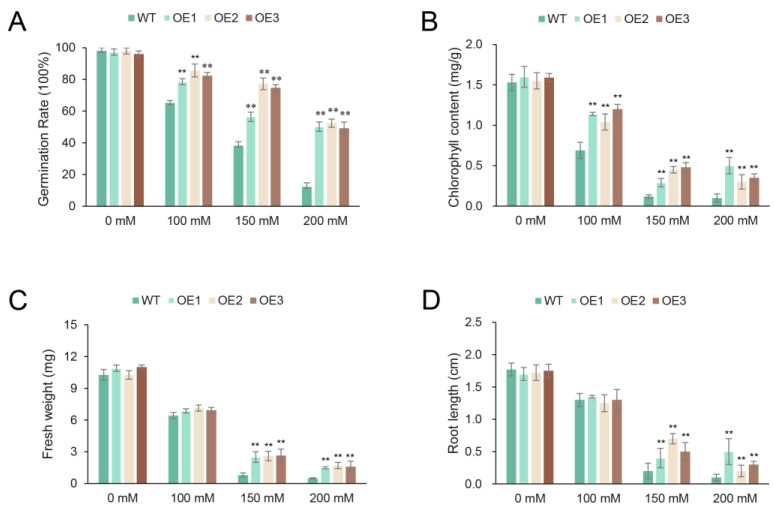
Physiological characteristics including germination rate (**A**), chloroplast content (**B**), fresh weight (**C**), and root length (**D**), of 10-day-old Arabidopsis seedlings that over-expressed *SaR2R3-MYb15* under the control or stress conditions (100, 150, and 200 mM NaCl). Values are the mean ± SD of three replicates. The symbol ** stands for *p* < 0.01.

To determine the function of *SaR2R3-MYB15* in response to salt stress, leaves from three different homozygous T3 transgene-positive Arabidopsis lines and wild-type seedlings were used to assay the chloroplast content, germination rate, fresh weight, and root length. Under the controlled conditions, all transgene-positive lines and wild-type seedlings were grown on half-strength MS agar plates with different concentrations of NaCl (0, 100, 150, and 200 mM) for 10 days. Our results showed no significant differences between transgene-positive lines and wild-type plants under normal growth without salt treatment. Although the transgenic lines and wild-type plants could grow on 100, 150, or 200 mM NaCl-treated MS agar plates, the wild-type plants had a lower germination rate (Figure 8A), chloroplast content (Figure 8B), and fresh weight (Figure 8C), as well as a shorter root length (Figure 8D), compared with the transgenic lines.

### 2.8. Overexpression of SaR2R3-MYB15 Enhanced the Salt Tolerance by Altering the Ability to Scavenge Reactive Oxygen Species (ROS)

**Figure 9:**
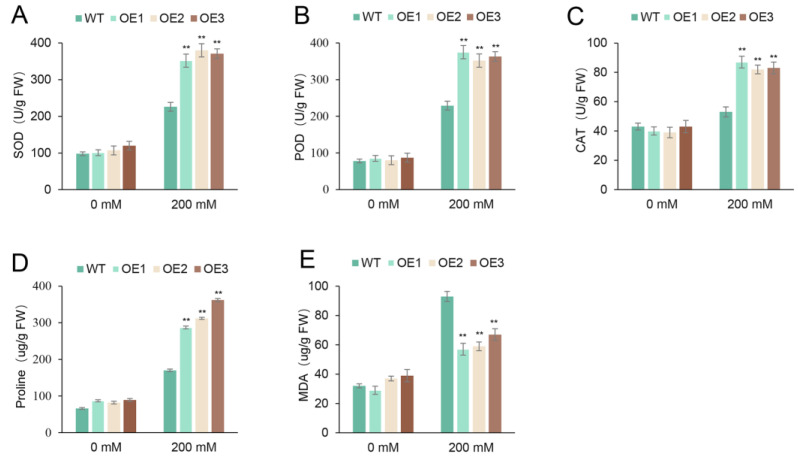
The antioxidant enzyme activity including superoxide dismutase (SOD) (**A**), peroxidase (POD) (**B**), catalase (CAT) (**C**), and the contents of proline (**D**) and malondialdehyde (MDA) (**E**) were measured with 10-day-old Arabidopsis seedlings. Values are the mean ± SD of three replicates. The symbol ** stands for *p* < 0.01.

The activities of different antioxidative enzymes (SOD, POD, and CAT) and the contents of MDA and proline were used to evaluate the salt tolerance imbued by scavenging ROS in transgenic lines. The activities of SOD, POD, and CAT were similar between transgenic lines and wild-type seedlings under normal conditions (Figure 9A–C). Under salt stress, both transgenic lines and wild-type seedlings exhibited increased activities of these enzymes, but the increase was particularly remarkable in the different transgenic lines (Figure 9D,E). Similarly, the proline content also increased in the transgenic lines and wild-type seedlings, with a more pronounced increase observed in the different transgenic lines. The MDA content of transgenic lines and wild-type seedlings under salt treatment was higher than that under normal growth conditions. Notably, the concentration of MDA was significantly higher in wild-type seedlings compared to transgenic lines. These findings indicated that overexpressed *SaR2R3-MYB15* could mitigate ROS accumulation and reduce cell membrane damage in transgenic Arabidopsis under salt stress.

### 2.9. Overexpression of SaR2R3-MYB15 Increased the Expression of Salt Stress-Related Genes

**Figure 10:**
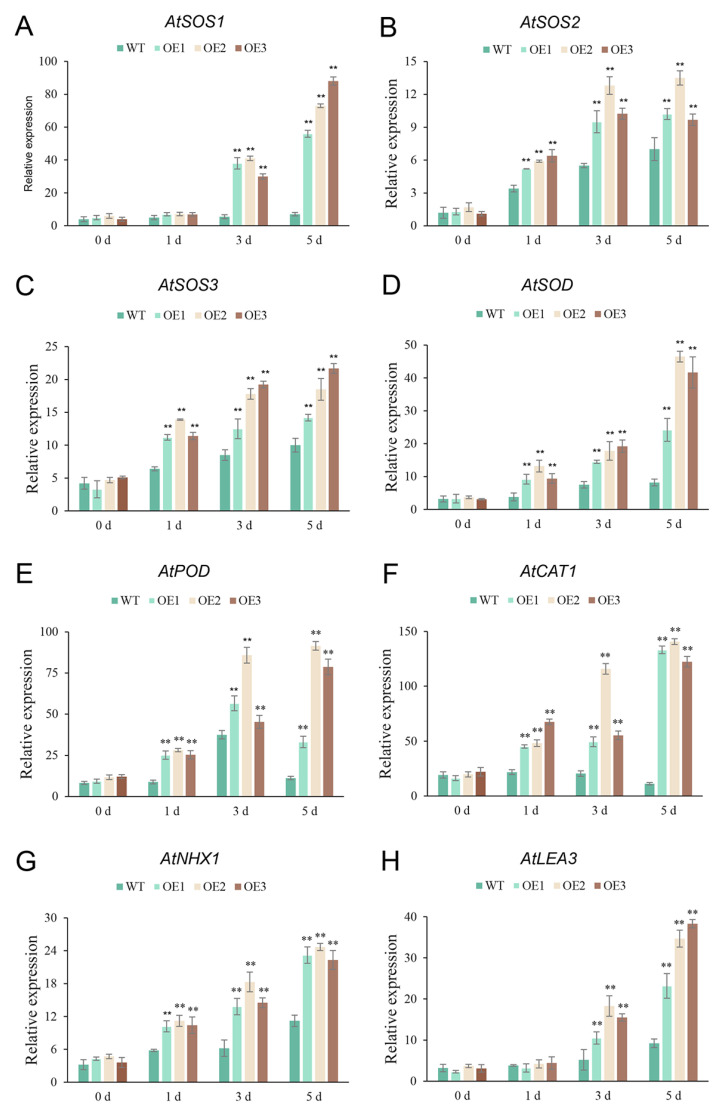
The expression of several genes including *AtSOS1* (**A**), *AtSOS2* (**B**), *AtSOS3* (**C**), *AtSOD* (**D**), *AtPOD* (**E**), *AtCAT1* (**F**), *AtNHX1* (**G**), and *AtLEA3* (**H**) in 40-day-old Arabidopsis seedlings overexpressing *SaR2R3-MYB15*, as well as in wild-type seedlings, under 0, 1, 3, and 5 days of salinity stress. Values are the mean ± SD of three replicates. The symbol ** stands for *p* < 0.01.

To investigate the underlying mechanism of enhanced salt tolerance in transgenic lines, seven known Arabidopsis stress-response genes associated with osmotic and oxidative stress were examined, including *AtNHX1*, *AtSOD*, *AtPOD*, *AtCAT1*, *AtSOS1*, *AtSOS2*, and *AtSOS3* (Figure 10). Comparative analysis revealed significantly higher expression levels of these genes in transgenic lines than in wild-type seedlings when subjected to 100, 150, and 200 mM NaCl concentrations. The findings indicated that *SaR2R3-MYB15* could enhance salt tolerance by increasing the expression of salt stress-related genes.

## 3. Discussion

### 3.1. Characteristics of SaR2R3-MYB TFs in S. alopecuroides

Higher plants have devolved various mechanisms to cope with salt stress, including activating specific transcriptional factors and changing the expression of related genes [8,10]. The R2R3-MYB transcription factors are crucial for regulating plant growth and development, cell differentiation, secondary metabolism, and stress responses [2,3]. Extensive studies have been conducted on the R2R3-MYB gene families in plants, using functional analysis and genome-wide bioinformatics approaches [3,4]. Although *S. alopecuroides* is a leguminous perennial plant with a high tolerance to stress and is known to be a potential source of stress resistance genes, few systematic investigations have been carried out to identify the R2R3-MYB gene family and explore potential functions in response to salt stress. In this study, the number of SaR2R3-MYB TFs identified in *S. alopecuroides* was lower than that in species with complete genome sequencing by genome-wide identification, such as *Arabidopsis* [4], rice [6], and soybean [23]. Likewise, the number of SaR2R3-MYB TFs in *S. alopecuroides* was somewhat smaller than that in *Hordeum vulgare* (51) [24], *Cryptomeria fortune* (35) [25], and *Ginkgo biloba* (45) [26], in a manner comparable to the transcriptome-wide identification of R2R3-MYB. The variation in the numbers of R2R3-MYB TFs among different species, including the relatively small number found in *S. alopecuroides*, could be attributed to species-specific evolutionary differences, or to the limitations of the data provided by the transcriptome in this study. Future research should conduct protein analysis based on data from whole-genome sequencing investigations to obtain more accurate insights.

The conserved amino acids of the TFs were remarkably similar between *S. alopecuroides* and *Arabidopsis*, indicating the high conservation of this domain across plant MYB genes. The third helix of the MYB domain was found to contain most of the other conserved residues, suggesting a higher degree of conservation than helices 1 and 2. The sequences of helix 3 in *S. alopecuroides* were very similar to one another, suggesting a similar function. Furthermore, there was significant variation in the amino acid type and length, molecular weights, and isoelectric point in *S. alopecuroides*, which could be attributed to an evolutionary event, such as exon insertion or loss, functional diversity, and sub-functionalization within different subfamilies [27,28].

### 3.2. Phylogenetic Analysis and Functional Prediction of SaR2R3-MYB TFs

Phylogenetic analysis revealed that R2R3-MYBs within the same branch exhibit similar sequences, conserved motifs, and biological functions [29,30]. Therefore, phylogenetic analysis combined with comparisons to annotated proteins has proven to be a practical approach for inferring the functions of unannotated proteins. The phylogenetic tree showed that there were species-specific subgroups that only contained SaR2R3-MYB proteins from *S. alopecuroides*, indicating that R2R3-MYB TFs had undergone duplications following lineage divergence and still exhibited evolutionary conservation characteristics. Furthermore, based on homology, the phylogenetic tree provided useful information about the potential functions, especially for R2R3-MYB TFs clustered in the same evolutionary branch. For instance, there were three SaR2R3-MYB TFs that were grouped with AtMYB11, AtMYB12, and AtMYB111 in cluster S7, which are known to play significant roles in flavonoid biosynthesis [31,32]. Therefore, it was reasonable to assume that these three TFs from *S. alopecuroides* could also be involved in flavonoid metabolism. Notably, it was demonstrated that AtMYB111 regulates salt responses through the modulation of flavonoid biosynthesis in *Arabidopsis* [33]. This finding indicated that TFs within this cluster (S7) could contribute to salt-stress responses by promoting the accumulation of flavonoids.

Similarly, three AtR2R3-MYB TFs and two SaR2R3-MYB TFs were grouped into cluster S2. Previous research highlighted that AtR2R3-MYB13 plays a regulatory role in meristem function [34] and contributes to boric acid tolerance [35]. AtR2R3-MYB14 changes the expression of CBF genes to control freezing tolerance [36]. AtR2R3-MYB15 was shown to enhance the expression of stress-inducible genes, including those involved in salt stress and abscisic acid (ABA) biosynthesis and signaling [22]. Based on these findings, it was reasonable to infer that the SaR2R3-MYB TFs grouped within the same clade as their *Arabidopsis* counterparts could share similar functions and contribute to multiple biotic stress responses.

### 3.3. Expression Patterns and Potential Function of the SaR2R3-MYB TFs in Salt Stress

Gene expression profiling provides valuable insights into the functional characterization of genes and the complex regulatory pathways involved in responses to biotic and abiotic stress [37]. The R2R3-MYB TFs, with their highly diverse DNA-binding domains, are known to play essential roles [38]. Our study revealed that most SaR2R3-MYB TFs showed high expression profiles under salt stress. In *Arabidopsis*, multiple TFs, including AtMYB12 [39], AtMYB15 [22], AtMYB20 [13], AtMYB42 [14], AtMYB44 [40], and AtMYB74 [41], were proven to regulate the expression of salt-, hormone-, or dehydration-responsive genes to enhance the tolerance to salt stress. Recent research showed that the salt overly sensitive (SOS) genes are suppressed by AtMYB73, which functions as a negative regulator of salt responses [42]. According to the phylogenetic analysis and protein–protein interaction network, we found the SaR2R3-MYB15 had a close relationship with AtMYB15, a transcription factor induced by salt stress. Based on the transcriptome data, *SaR2R3-MYB15* also showed a high level of gene expression, and its expression was more than 10 times higher under salt stress than in the control group, which was further conformed by the qRT–PCR analyses. Hence, we speculated that SaR2R3-MYB15 was crucial in enhancing salt tolerance in *S. alopecuroides*.

Salt stress in plants leads to the overproduction of reactive oxygen species (ROS), affecting the levels of proteins, fluids, and photosynthetic pigments [43]. Plant antioxidant systems respond to salt stress by modulating the activities of enzymatic antioxidants, such as superoxide dismutase (SOD), peroxidase (POD), and catalase (CAT), to protect cells from oxidative damage [44]. Our study showed a significant increase in the activity of SOD, POD, and CAT in transgenic Arabidopsis plants under high-concentration saline growth conditions. Moreover, the transgenic lines exhibited higher proline accumulation than wild-type plants. The transgenic lines also showed lower malondialdehyde (MDA) levels, indicating reduced oxidative damage and superior growth compared to wild-type plants. Additionally, the overexpression of *SaR2R3-MYB15* in tobacco resulted in decreased levels of MDA compared to the controls. These physiological changes suggest a potential role for *SaR2R3-MYB15* in enhancing salt-stress tolerance.

The SOS signaling pathway and ion homeostasis regulation play significant roles in cellular salt tolerance. Overexpression of NHX transporters enhances salt-stress tolerance in Arabidopsis [45]. Plants accumulate LEA proteins under contrasting salt tolerance [46]. Overexpression of the strawberry *FvMYB24* in Arabidopsis induces the upregulation of POD, SOD, and CAT and improves salt-stress tolerance [47]. Similarly, overexpressing *SaR2R3-MYB15* in Arabidopsis led to the upregulation of eight stress-related genes, thereby conferring salt tolerance. Moreover, the expression levels of salt-related genes were dramatically increased by temporarily overexpressing *SaR2R3-MYB15* in tobacco. Similarly, overexpressing three *Osmanthus fragrans* TFs in tobacco resulted in a dramatic increase in the expression of stress-related genes, indicating their significant roles in salt stress [48]. 

Therefore, the salt-inducible SaR2R3-MYB TFs could enhance salt tolerance in transgenic plants. These findings might be important for understanding the regulatory roles of SaR2R3-MYB TFs in response to salt stress, while further research is required to elucidate the molecular mechanisms underlying salt stress in *S. alopecuroides*.

## 4. Materials and Methods

### 4.1. Identification of SaR2R3-MYB TFs

The transcriptomes of *S. alopecuroides* leaves under salt stress [16] (BioProject accession number: PRJNA748576) were used to investigate the R2R3-MYB gene family and study their gene expression patterns. Clean reads were obtained after eliminating low-quality raw data using fastp software [49] with default parameters. As no reference genome was available for *S. alopecuroides*, de novo transcriptome assembly was performed using Trinity v. 2.7.0 software with default settings (k-mer size = 25) [50]. TransDecoder v. 5.5.0 [50] was utilized to predict the coding regions of the assembled transcripts, and transcripts with more than 100 peptides were selected to identify the single best open reading frame (ORF). To minimize redundancy, CD-HIT-EST software [51] with default parameters (95% similarity) was employed to generate unique genes, referred to as “unigenes”.

For the identification of R2R3-MYB family members, the Hidden Markov Model profile of the MYB domain (PF00249) was obtained from the InterPro database [52] and used to search against the *S. alopecuroides* protein databases. The BLASTP tool was applied to identify possible SaR2R3-MYB proteins in *S. alopecuroides* using Arabidopsis R2R3-MYB proteins as query sequences, acquired from PlantTFDB (http://planttfdb.gao-lab.org/, accessed on 20 January 2023). Subsequently, candidate SaR2R3-MYB proteins were evaluated using the SMART database [53]. Additionally, the properties of SaR2R3-MYB proteins were examined using the ExPASy server [54].

### 4.2. Phylogeny and Conserved Domain Protein Sequence Analysis of SaR2R3-MYB TFs

To validate and visualize the conserved domains of SaR2R3-MYB proteins, multiple sequence alignments were performed using MUSCLE v5.0 software (https://www.drive5.com/muscle/, accessed on 20 January 2023). The alignment results were visualized using WebLogo v2.8.2 software (https://weblogo.berkeley.edu/, accessed on 20 January 2023). A phylogenetic tree was constructed using the neighbor-joining method with SaR2R3-MYB proteins and AtR2R3-MYB proteins, using MEGA v7.0 software [55]. Evolview v3.0 [56] was used to display the phylogenetic tree.

### 4.3. Protein–Protein Interaction Network Prediction

Protein–protein interaction network analyses were conducted using the STRING database (https://string-db.org/, accessed on 20 January 2023) to investigate the regulatory interactions of 28 SaR2R3-MYB protein sequences from *S. alopecuroides* and their corresponding orthologs from Arabidopsis. The regulatory interactions were visualized using Cytoscape v3.0 (https://cytoscape.org/cy3.html, accessed on 20 January 2023).

### 4.4. Plant Materials and Treatments

Sixty-day-old *S. alopecuroides* seedlings were cultivated in a greenhouse as plant material at Nanjing Forestry University (Nanjing, China). Seedlings with similar developmental patterns were potted and placed in a growth chamber for three weeks under the following conditions: day/night temperatures of 25 °C/20 °C, relative humidity of 58%, a photoperiod of 16 h light and 8 h dark, and a light intensity of 260 μmol m^−2^ s^−1^.

The seedlings were subjected to NaCl solutions with concentrations of 50, 150, and 200 mM to induce salt stress. These solutions were mixed half-and-half with Hoagland’s nutritional solution. After exposure to the salt-stress treatment, samples were collected at three time points: 0, 3, and 7 days. The entire experiment was performed using three biological replicates.

### 4.5. Transcriptome Analysis and Verification by Quantitative Real-Time PCR (qRT–PCR)

In accordance with previous reports, *S. alopecuroides* leaves subjected to 150 mM NaCl-induced salt stress for 0 days (S1), 3 days (S2), and 7 days (S3) were selected to perform transcriptome sequencing [16]. High-quality clean reads obtained from the transcriptomes of *S. alopecuroides* leaves under salt stress were used to explore the gene expression profiles of SaR2R3-MYB TFs, using Trinity software with default parameters [16]. The RPKM (reads per kb per million reads) of each unigene was determined and used to quantify gene expression levels [57]. Differential expression analysis was conducted using the edgeR program [58] with the selection criteria of absolute fold change ≥ 2 and corrected *p*-value ≤ 0.05.

Total RNA was extracted from the samples using a plant RNA extraction kit (TIANGEN, Beijing, China). Subsequently, cDNA synthesis was carried out using the PrimeScript™ RT master mix (TaKaRa, Beijing, China). The qRT–PCR was performed according to a previously described protocol [20]. Biological triplicates were used for each reaction, and the relative gene expression was calculated using the 2^−ΔΔCT^ method. The internal reference gene was *SAc1* (J01298) [20]. The primer sequences for qRT–PCR were designed using Primer Premier 3.0 (https://primer3.ut.ee/, accessed on 20 January 2023) and are listed in Table S4.

### 4.6. Subcellular Localization and Transcriptional Activation Analyses of SaR2R3-MYB15

To determine the subcellular localization, we amplified the coding sequence of *SaR2R3-MYB15* without a stop codon from *S. alopecuroides*, and then we cloned it into the pCAMBIA3301 along with the green fluorescent protein (GFP), generating the 35S::SaR2R3-MYB15-GFP fusion construct. We subsequently infiltrated tobacco (*Nicotiana benthamiana*) leaf tissue with Agrobacterium tumefaciens GV3101, which carried either the recombinant vector (35S::SaR2R3-MYB15-GFP) or the control vector. Tobacco leaf tissue was also infiltrated with the Agrobacterium tumefaciens GV3101 carrying the recombinant vector (35S::SaR2R3-MYB15-GFP) and the control vector. The GFP signal was observed using a confocal microscope (TCS-SP8 Leica, Wetzlar, Germany).

The full-length coding sequence of *SaR2R3-MYB15* was amplified and integrated into pGBKT7 (Contech) to explore its transcription activation. The vector was then transformed to yeast strain *Saccharomyces cerevisiae* AH109 (Coolaber, Beijing, China) and cultured at 30 °C for 2–3 days on the SD/-Trp, SD/-Trp-His-Ade, and SD/-Trp-His-Ade with X-α-gal selective media. All primer sequences used to perform subcellular localization and transcriptional activation analyses are listed in Table S4.

### 4.7. Transient Transformation of SaR2R3-MYB15 into Tobacco and Measurement of Malondialdehyde (MDA) Levels

After being introduced into *A. tumefaciens* GV3101, the pCAMBIA1300 (control) and pCAMBIA1300-SaR2R3-MYB15 fusion vectors were integrated into 4-week-old leaves of tobacco plants using the Agrobacterium-mediated transient transformation. The transiently transformed tobacco was cultivated under the following conditions in the growth chamber: relative humidity of 60%, day/night temperatures of 25 °C/20 °C, a photoperiod of 16/8 h light/dark, and a light intensity of 260 μmol m^−2^ s^−1^. After two days of cultivation, the tobacco plants were irrigated with either 0 or 200 mM NaCl. Samples were collected after 1, 3, and 5 days, with three biological replicates for each treatment. The MDA content was determined according to the instructions provided by the MDA kit (Nanjing Jiancheng Biological Engineering Institute, Nanjing, China).

### 4.8. Genetic Transformation of SaR2R3-MYB15 in Arabidopsis and Determination of Physiological and Biochemical Parameters

The fusion vector pCAMBIA1300-SaR2R3-MYB15 was utilized to transform into Arabidopsis via the Agrobacterium-mediated stable transformation method. We selected the positive transgenic lines from the T0 seedlings that were grown and germinated on MS medium containing 25 mg/L hygromycin for 15 days. The positive homozygous T3 progenies were then selected for further analysis.

A total of 100 Arabidopsis seeds of transgenic lines and wild-type seedlings under salt stress (0, 100, 150, and 200 mM NaCl) were used to perform germination assays. We defined germination according to the principle that there was an obvious emergence of the radicle through the seed coat after 2 days of stratification at 4 °C. The fresh weight and root length of germinated seeds were measured after cultivation for 10 days. Moreover, the total chlorophyll content was also determined using the biochemical kit (Solarbio Life Science, Beijing, China). Additionally, referring to the corresponding kit instructions (Nanjing Jianchen Biological Engineering Institute, Nanjing, China), the antioxidant enzyme (CAT, POD, and SOD) activities and the contents of proline and MDA were measured using the transgenic plants and wide-type seedlings under salt stress (200 mM NaCl).

After sterilization, seeds of transgenic lines and wild-type seedlings were cultured on 1/2 MS medium at 22 °C with a 16 h light/8 h dark cycle in a growth chamber. Twenty-day-old seedlings with similar growth potential were grown in plastic plots with a mixture of organic soil and sand (3:1, *v*/*v*). The culture conditions of Arabidopsis were similar to those mentioned for tobacco in a greenhouse. Arabidopsis plants were irrigated with 0 and 200 mM NaCl solution every two days. After irrigation with saline water 3 times, samples were collected at the designated times (0, 1, 3, and 5 days), with three biological replicates, for salt stress-related gene expression analysis.

### 4.9. Gene Expression Analysis of Salt Stress-Related Genes in Transgenic Plants

Total RNA was extracted from the collected leaf samples using a plant RNA extraction kit (TIANGEN, Beijing, China), following the manufacture’s instructions. The expression patterns of salt stress-related genes (*NtSOD*, *NtCAT*, and *NtAPX*) were investigated in the transgenic tobaccos and the control groups were investigated. The *NbL25* was used as an internal reference in the qRT–PCR analysis [48]. Additionally, we examined the expression patterns of eight salt stress-related genes (*AtSOS1*, *AtSOS2*, *AtSOS3*, *AtSOD*, *AtPOD*, *AtCAT1*, *AtNHX1*, and *AtLEA3*) in both the transgenic Arabidopsis and control groups. The internal control gene *AtActin* was selected for normalization. The primers used for amplification are listed in Table S2.

## 5. Conclusions

The transcriptome analysis of *S. alopecuroides* under salt stress led to the identification and characterization of 28 SaR2R3-MYB TFs. Sequence alignments revealed a conserved R2R3 domain with three regular tryptophan helices. Phylogenetic analysis and functional predictions suggested that some SaR2R3-MYB TFs likely played crucial roles in controlling various biological processes. Distinct expression profiles of SaR2R3-MYB TFs were observed under salt stress, and the protein–protein interaction network suggested unique functions for certain proteins in response to salt stress. Additionally, our investigations showed that SaR2R3-MYB15 localized to the nucleus and displayed transcription activity. Transient transformation of tobacco and genetic transformation of Arabidopsis with *SaR2R3-MYB15* resulted in enhanced salt tolerance, as evidenced by changes in the activity of antioxidative enzymes and the contents of profile and MDA. Furthermore, the overexpression of *SaR2R3-MYB15* could also increase the expression of several stress-related genes to enhance the tolerance of transgenic plants. This discovery provides a solid foundation for understanding the molecular mechanisms of SaR2R3-MYB TFs in response to salt stress in *S. alopecuroides.*

## Data Availability

The article contains all the information required to support its conclusions.

## Supplementary Materials

The following supporting information can be downloaded at: https://www.mdpi.com/article/10.3390/plants13050586/s1, Figure S1: The expression profiles of SaR2R3-MYB genes under salt stress after 1, 3, and 5 days were examined using a qRT–PCR assay. Student’s test was used to assess significant differences and the standard errors are shown as the value of the error bars (**, *p* < 0.01). Figure S2: The contents of MDA in three different tobacco strains at different time points under salt stress. L1–3 and L4–6 represent the control group and three different tobacco strains injected with pCAMBIA1301-SaR2R3-MYB15, respectively (A). The MDA contents in tobacco transiently transformed with the SaR2R3-MYB15 gene under salt treatment after 1, 3, and 5 days (B). Table S1: Detailed information about the CDS and protein sequences of SaR2R3-MYB in *S. alopecuroides*. Table S2: Detailed information about the physicochemical properties of SaR2R3-MYB genes in *S. alopecuroides*. Table S3: Gene Ontology results in protein–protein pathways. Table S4: Different primer sequences used in the study.
