# Supplementary figures and images for: Transcriptome-Based Identification of the SaR2R3-MYB Gene Family in Sophora alopecuroides and Function Analysis of SaR2R3-MYB15 in Salt Stress Tolerance
Source: Plants (Basel). 2024 Feb 21;13(5):586. doi: 10.3390/plants13050586 (PMC10934591; doi:10.3390/plants13050586)

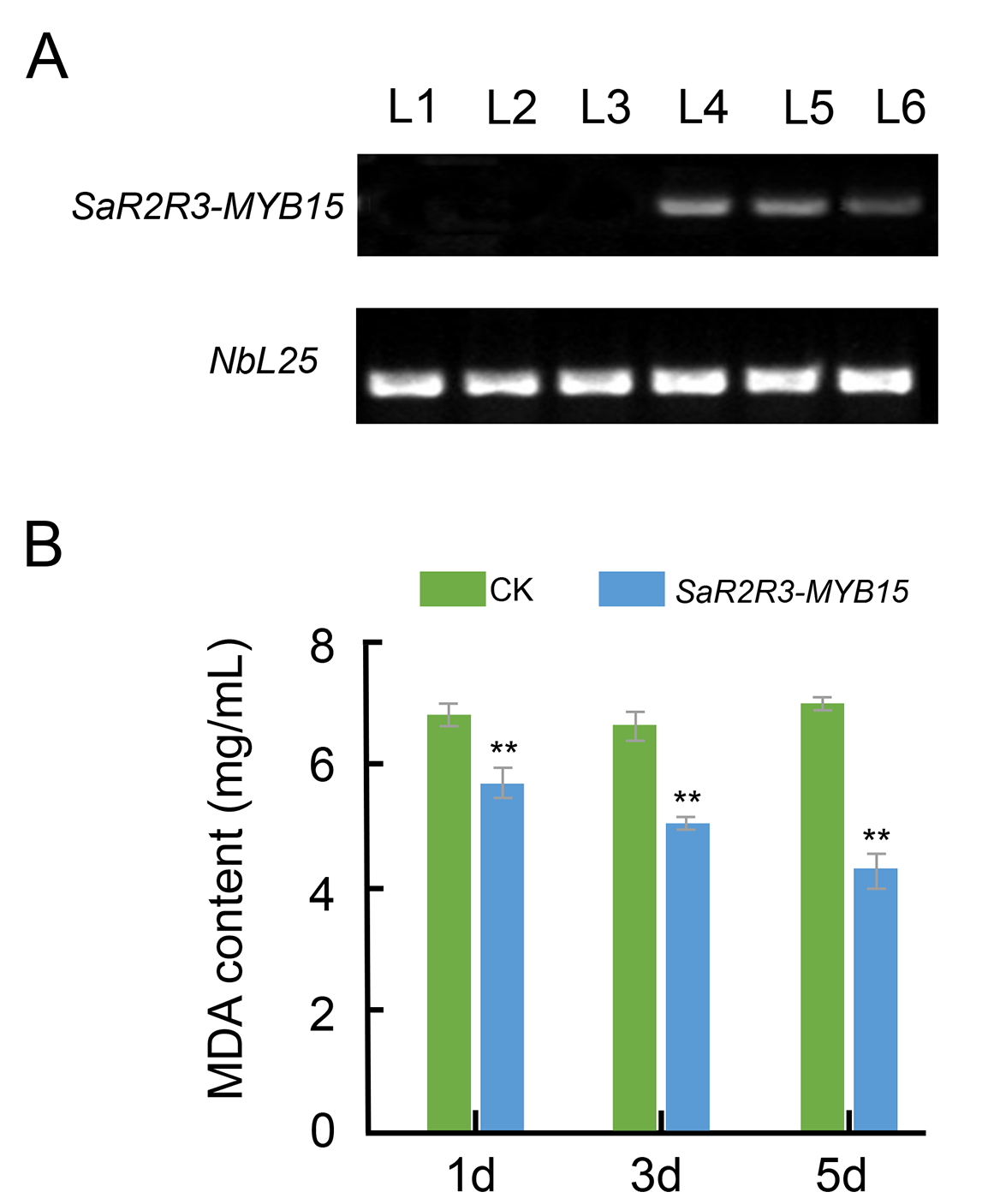

Supplement: Supplementary file 1 [file plants-13-00586-s001.zip › Supplementary Figure 2.tif]

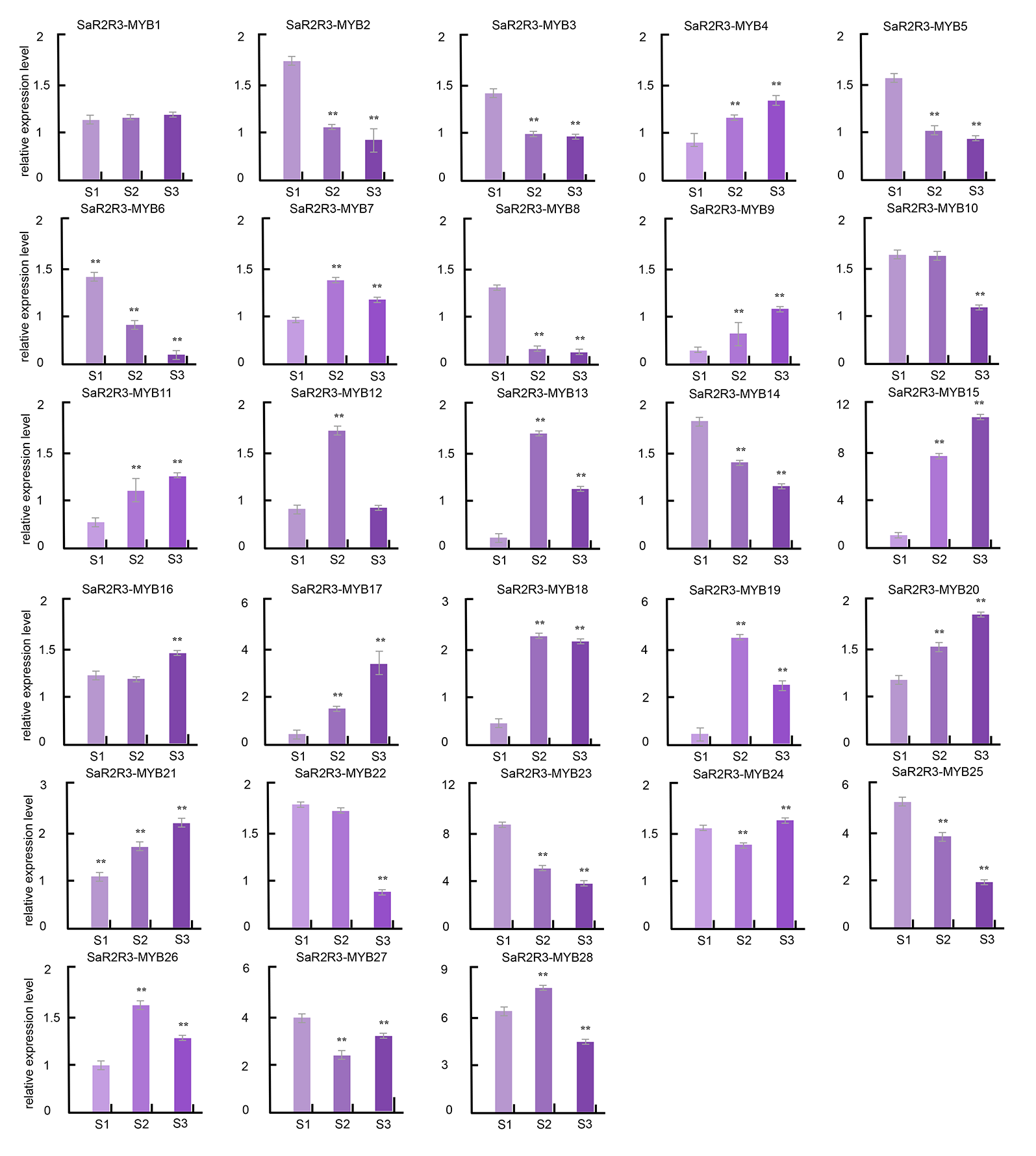

Supplement: Supplementary file 1 [file plants-13-00586-s001.zip › Supplementary Figure 1.tif]
